# The Mitochondrial Genome of *Cylicocyclus elongatus* (Strongylida: Strongylidae) and Its Comparative Analysis with Other *Cylicocyclus* Species

**DOI:** 10.3390/ani12121571

**Published:** 2022-06-17

**Authors:** Yuan Gao, Zhonghuai Zhang, Chunren Wang, Kai Zhao

**Affiliations:** 1 Institute of Nanobiomaterials and Immunology, School of Life Science, Taizhou University, Taizhou 318000, China; gaoyuan@tzc.edu.cn; 2 College of Animal Science and Veterinary Medicine, Heilongjiang Bayi Agricultural University, Daqing 163319, China; zhonghuaizhang@126.com (Z.Z.); chunrenwang@sohu.com (C.W.)

**Keywords:** *Cylicocyclus elongatus*, mitochondrial genome, comparative analyses, phylogenetic analyses

## Abstract

**Simple Summary:**

We first report the complete mitochondrial genome of *C. elongatus*, which is circular and 13,875 bp in size, containing 12 PCGs, 22 tRNAs, 2 RNAs, and 2 NCRs. Comparative analyses and phylogenetic analyses show that *C. elongatus* is a member in *Cylicocyclus* based on mt genome data.

**Abstract:**

*Cylicocyclus elongatus* (*C. elongatus*) is one of the species in *Cylicocyclus*, subfamily Cyathostominae, but its taxonomic status in *Cylicocyclus* is controversial. Mitochondrial (mt) genome is an excellent gene marker which could be used to address the taxonomy controversy. In the present study, the complete mt genome of *C. elongatus* was determined, and sequence and phylogenetic analyses were performed based on mtDNA data to determine the classification of *C. elongatus*. The circular complete mt genome of *C.* *elongatus* was 13875 bp in size, containing 12 protein-coding genes (12 PCGs), 22 transfer RNA (tRNA) genes, 2 ribosomal RNA (rRNA) genes, and 2 non-coding regions (NCRs). The A + T content of *C. elongatus* complete mt genome was 76.64%. There were 19 intergenic spacers with lengths of 2–53 bp and 2 overlaps with lengths of 1–2 bp in the impact complete mt genome. ATT and TAA were the most common start and termination codons of 12 PCGs, respectively. Comparative analyses of mt genomes nucleotide sequence and amino acid sequence showed that there were higher identities between *C. elongatus* and five other *Cylicocyclus*, rather than with *P. imparidentatum*. Phylogenetic analyses based on concatenated nucleotide sequences of 12 PCGs of 23 species in the family Strongylidae showed that *C. elongatus* was closely related to *Cylicocyclus* species, rather than *P. imparidentatum*. We concluded that *C. elongatus* was a member in *Cylicocyclus* based on comparative and phylogenetic analyses of mt genome sequences. The data of the complete mt genome sequence of *C. elongatus* provide a new and useful genetic marker for further research on Cyathostominae nematodes.

## 1. Introduction

Cyathostominae (Strongylida: Strongylidae) nematodes are a group of significant pathogens of equines which inhabit the large intestine [1]. There are more than 50 species in the subfamily, and the occurrence of co-infection of different kinds of parasites is quite common in hosts [2]. Co-infection of these adult parasites can cause dropsy, diarrhoea, and weight loss, and the larvae can cause fatal cyathostominosis [3,4,5].

Cyathostomins are considered to be one of the primary parasites of equids. The taxa controversies in the subfamily have been discussed for at least one century. Researchers have primarily presented their views on the classification at the generic level of these nematodes [6,7]. *Cylicocyclus* Ihle, 1922 is the largest genus of Cyathostominae, and the taxonomic status of some species in the genus is still controversial [8]. *C. elongatus* is one of the members in the genus, which has a greatly elongated bursa and differs from congeners [1]. Phylogenetic analyses combining internal transcribed spacers (ITS)-1 and ITS-2 data suggested that *C. elongatus* clustered with the comparative species (*Petrovinema poculatum* and *Poteriostomum imparidentatum*), indicating that *C. elongatus* represents members of other genera [8].

Mt genomes have been successfully applied in parasite taxonomy, population genetics, and systematics, because of characteristics such as strict maternal inheritance and comparatively conserved genomic structure [9,10]. In previous studies, mt genomes have been used to address a lot of controversies on the status of certain taxa, such as the taxonomy of *Triodontophorus*, the identification of species complex, etc. [11,12,13]. Now, 13 complete mt genomes of Cyathostominae nematodes have been determined, including 5 complete mt genomes in *Cylicocyclus* [10,11,12,14]. However, the complete mt genome of *C. elongatus* has not been completed. Thus, to identify the classification of *C. elongatus*, the aims of the present study were to sequence the complete mt genome of *C. elongatus*, to compare the obtained complete mt genome sequences among congeneric species, and to reconstruct the phylogenetic relationships to assess the status of *C. elongatus* within the Cyathostominae.

## 2. Materials and Methods

### 2.1. Parasites and Molecular Identification of Specimens

Adult nematodes of *C. elongatus* were collected from naturally infected horses from a slaughterhouse in Daqing, Heilongjiang Province, China. The nematode was identified to the species level based on morphological features and molecular biology techniques [1]. Total genomic DNA was extracted from one adult *C. elongatus* by the TIANamp Genomic DNA Kit according to the manufacturer’s instructions (TIANGEN Biotech, Beijing, China) and stored at −20 °C until use. The ITS of *C. elongatus* were amplified for the identification of molecular biology using universal primers NC5 (5′-GTA GGT GAA CCT GCG GAA GGA TCA TT-3′) and NC2 (5′-TTA GTT TCT TTT CCT CCG CT-3′), reported previously [15].

### 2.2. The Amplification and Annotation of C. elongatus Complete Mt Genome

The primers of the complete mt genome amplification in this study were designed according to the mt genomes sequences of the congenic species. The conservative sequences of each region in those of congener species were selected to use as the candidate primers. The evaluation of these primers was performed using Oligo 6.0. Eight primers were used to amplify the complete mt genome as 8 overlapping fragments from genomic DNA (Table S1). PCR was conducted in a 50 µL reaction mixture containing 1 × Ex Taq Buffer, 0.2 mM dNTP Mixture, 0.625 U TaKaRa Ex Taq, 0.4 µM of each primer, and 1 µL gDNA under the following condition: an initial denaturing at 94 °C for 5 min, followed by 35 cycles (denaturing at 94 °C for 1 min, annealing at 55–40 °C for 30 s, and extension at 72 °C for 1–2 min), and a final extension at 72 °C for 7 min. All positive amplicons were purified, cloned into pMD 18-T vector, and transformed into *E. coli* DH5α. The positive clones were sent to Sangon Biotech Co., Ltd. (Shanghai, China) for sequencing.

The complete mt genome of *C. elongatus* was aligned with those of Cyathostominae nematodes [11,14,16]. Gene boundaries including 12 PCGs, 22 tRNAs, and 2 rRNAs were determined by the species in *Cylicocyclus* using Clustal X 1.83, MEGA X, and tRNAscan-SE 2.0 (tRNAscan-SE Search Server (ucsc.edu)) (accessed on 11 August 2020) [17,18].

### 2.3. Comparative Analyses of Cylicocyclus Species and P. imparidentatum Mt Genomes

Comparisons of *C. elongatus* complete mt genome with those of five species (*Cylicocylus ashworthi*, *Cylicocyclus insigne*, *Cylicocyclus radiatus*, *Cylicocyclus auriculatus*, and *Cylicocyclus nassatus*) in *Cylicocyclus* available in GenBank were performed, including lengths, identities, A + T contents, and protein codons [10,16,19]. Percentages of A + T content of each gene/region was computed using DNAStar (v. 12.1) [20]. GC and AT skews were calculated according to the following formulas: AT skew = (A − T)/(A + T) and GC skew = (G − C)/(G + C). The identities of amino acids and nucleotides sequences among 6 *Cylicocyclus* species and *P. imparidentatum* were calculated by MegAlign 5.01 [20].

### 2.4. Phylogenetic Analyses of 23 Strongylidae Nematodes

Phylogenetic analysis in the present study was based on the concatenated nucleotide sequences of 12 PCGs and complete mt genome nucleotide sequences of 23 Strongylidae nematodes available in GenBank, respectively. *Oxyuris equi* was used as the outgroup. The sequences of 12 PCGs of 23 Strongylidae nematodes were aligned using MAFFT 7.471 and then concatenated into a single alignment [21]. Sites of ambiguous alignment were eliminated using the online server Gblocks (Phylogeny.fr: Gblocks) (accessed on 12 October 2020). Maximum likelihood (ML) method was used to reconstruct the relationships by Mega X.

## 3. Results

### 3.1. The Annotation of C. elongatus Complete Mt Genome

**Figure 1:**
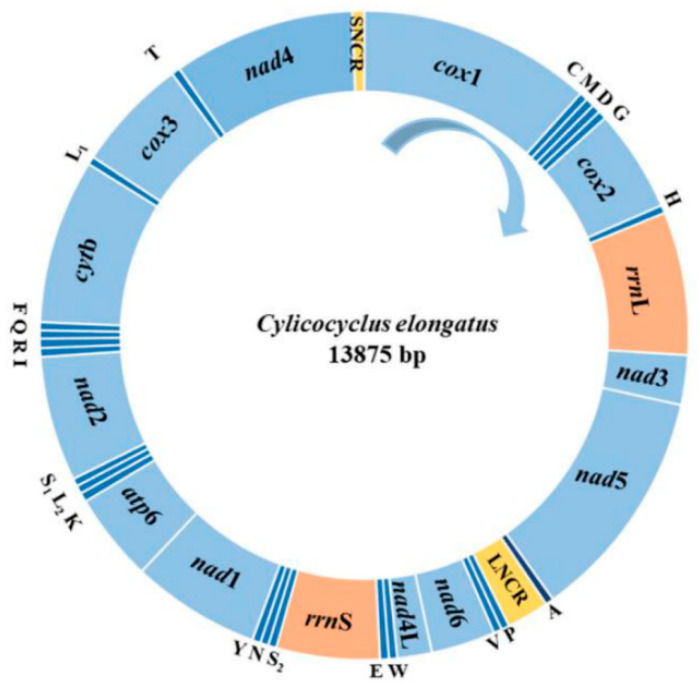
The composition, size, and transcription direction of *C. elongatus* complete mt genome.

The complete mt genome of *C. elongatus* was 13,875 bp in length. It contained 36 genes, including 12 PCGs (*cox*1-*cox*3, *cyt*b, *atp*6, *nad*1-*nad*6, and *nad*4L), 22 tRNAs, and 2 rRNAs (Table 1, Figure 1). The A + T and G + C contents of *C. elongatus* complete mt genome were 76.64% and 23.36%, with 0.39 and −0.19 AT skew and GC skew, respectively. These PCGs started at ATT and TTG, and stopped at TAA and TTG. *Cox*3 used the incomplete codon “T” as a stop codon. In the mt genome of *C. elongatus*, there were 19 spacer regions and two short overlaps. The spacer regions ranged from 2 bp to 52 bp in length, and the size of overlaps were all 1–2 bp.

There were two rRNAs (*rrn*L and *rrn*S) in the mt genome of *C. elongatus*, with lengths of 968 bp and 699 bp, respectively. The sizes of 22 tRNAs in the mt genome of *C. elongatus* were 53–63 bp. Moreover, a large NCR with a length of 286 bp and a shorter NCR with a length of 81 bp were found in the mt genome of *C. elongatus*. The A + T contents of the large and shorter NCRs were 85.31% and 81.48%, respectively.

### 3.2. Comparative Analyses of Mt Genomes among Cylicocyclus Species and P. imparidentatum

A comparison of amino acids and nucleotides sequence identities and lengths was made among six *Cylicocyclus* species and *P. imparidentatum* (Table 2 and Table S1). The identities of nucleotides sequences of the complete mt genomes among six *Cylicocyclus* species and *P. imparidentatum* were 82.9–90.7%. The identity of nucleotides sequence of complete mt genomes between *C. elongatus* and *P. imparidentatum* was 84.3%. These results showed that the identities between *C. elongatus* and *P. imparidentatum* were lower than those among *Cylicocyclus* species, indicating that *C. elongatus* had higher identities with congeneric nematodes (Table 2).

The lengths of 12 PCGs of *C. elongatus* were the same as those of other *Cylicocyclus* species, except for *cox*3 gene, which was 3 bp longer than that in the other five *Cylicocyclus* species. The size of *cox*3 in *C. elongatus* was longer than that in *P. imparidentatum* in Cyathostominae, but the size of *nad*4 was shorter (Table S1). As regards the identities of each mt gene, *P. imparidentatum* has lower identities to all five of the *Cylicocyclus* species, than those among *Cylicocyclus* species.

The A + T contents and skewness of *C. elongatus* were similar to those of *Cylicocyclus* species; however, the A + T content of 1st coding position of *C. elongatus* was higher than those in *Cylicocyclus* species and *P. imparidentatum*. The A + T skewness of first and second coding positions of *C. elongatus* was higher than those of *Cylicocyclus* species and *P. imparidentatum*, but the A + T skewness of third coding positions was lower than those of others in the present study (Figure S1).

### 3.3. Phylogenetic Analyses of 23 Species in the Family Strongylidae

**Figure 2:**
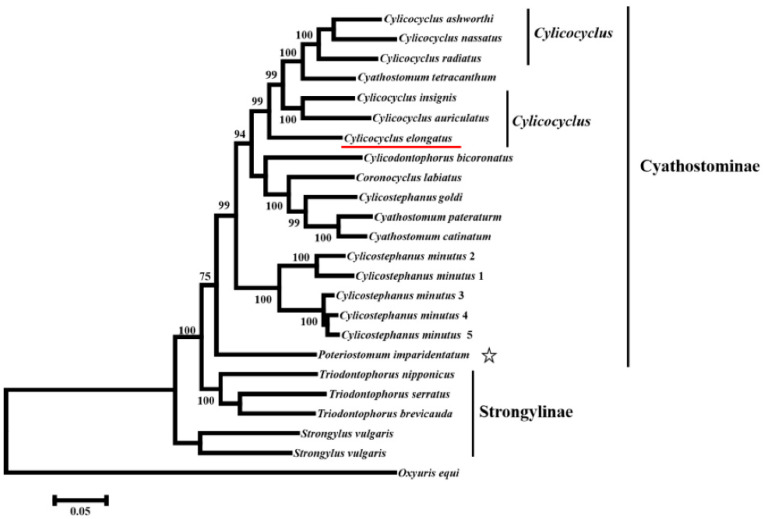
Phylogenetic analyses reconstructed using concatenated nucleotide sequences of 12 PCGs of complete mt genomes in 23 Strongylidae species. The tree was developed using ML method. *Oxyuris equi* is an outgroup. *C. elongatus* in current study is underlined.

In the current study, the phylogenetic relationship was reconstructed based on concatenated nucleotide sequences of 12 PCGs from 23 species in the family Strongylidae using ML method (Figure 2). The topological structure of the phylogenetic trees divided Strongylidae into two clades. One clade included 2 species, *Strongylus*, and another clade grouped 21 other species together in the family Strongylidae. *C.* *elongatus* and congeneric species are closely related to each other rather than to *P. imparidentatum*. Moreover, based on the tree in the current study, the results showed *C. elongatus* formed a distinct branch in the clade of *Cylicocyclus*, indicating that *C. elongatus* might be relatively closely related to other *Cylicocyclus* species that did not obtain complete mt genomes. The phylogenetic relationships reconstructed by nucleotide sequences of complete mt genomes of 23 species of the family Strongylidae were similar to those of Figure 2 (Figure S2). In the trees, *C. elongatus* was also closely related to congeneric species rather than to *P. imparidentatum.*

## 4. Discussion

The complete mt genome of *C. elongatus* was described first in the present study. Its composition, gene orders, transcription direction, and codon usages were the same as other Cyathostominae parasites [11,12,14]. Its sizes were slightly shorter than that of *C. ashworthi* (13,876 bp), longer than those of others in Cyathostominae, such as *P. imparidentatum*, *C. nassatus*, and *C. radiatus* [11,12,14]. The A + T contents of *C. elongatus* complete mt genomes were 76.64%, which was similar to those of nematodes such as *C. radiatus, Cy. catinatum*, and *Cs. minutus* [11,14]. The complete mt genome of *C.* *elongatus* had an impact structure that was similar to *C. insigne*, *C. radiatus*, and *P. imparidentatum* [10,11,14].

Comparison of nucleotide and amino acid sequences of mt genomes among six *Cylicocyclus* species and *P. imparidentatum* was performed. In previous studies, the identities of nucleotide sequences of complete mt genomes in congeners were 94.6% identity between *Cyathostomum pateratum* and *Cyathostomum catinatum*, 84.7% identity between *Chabertia ovina* and *Chabertia erschowi*, and 86.0% identity between *Triodontophorus serratus* and *Triodontophorus nipponicus*, respectively [22,23,24]. In the present study, *C. elongatus* had higher identities to congeneric nematodes rather than to *P. imparidentatum*, indicating that *C. elongatus* was more similar to *Cylicocyclus* species in terms of nucleotide sequences [11,14,16]. The results also indicated that *C. elongatus* might be a member to *Cylicocyclus.* AT bias in nucleotide composition of the complete mt genomes reflected a bias in the amino acid composition of proteins. Highly AT-rich regions for each genome may represent the origin of replication [10,25]. In the present study, higher A + T skewness of first and second coding positions were found in the complete mt genome of *C. elongatus* indicating that it might be more easily changed in the evolution.

In the current study, the phylogenetic relationships were reconstructed by nucleotide sequences. The topological structures of phylogenetic trees were similar with different data, which all supported that *C. elongatus* was closely related to *Cylicocyclus*, rather than to *P. imparidentatum*. The results were inconsistent with a previous study, which showed that *C. elongatus* was closely related to *P. imparidentatum*, *C. ultrajectinus*, and *P*. *poculatum* based on the combined ITS-1 and ITS-2 sequences [8]. The discrepancy was caused by the different gene markers used for evolutionary analysis. In many previous studies, it has been demonstrated that mt genomes are better gene markers for phylogenetic analyses, because of characteristics such as strict maternal inheritance, apparent lack of recombination, rapid evolutionary rate, and comparatively conserved genomic structure [9,10,25]. ITS, as the important region in rDNA, was more suitable for species identification. Though we did not obtain the complete mt genomes of *C. ultrajectinus* and *P*. *poculatum*, we also concluded that *C. elongatus* did not closely relate to *P. imparidentatum*, and *C. elongatus* did not represent members of other genera. We also expect to sequence more complete mt genomes in Cyathostominae to discuss and support these views. Moreover, for all trees in the present study, it is interesting that *Cyathostomum tetracanthum* arose within *Cylicocyclus*. The result was not in conformity with the classification based on morphology. More complete mt genomes of *Cy. tetracanthum* should be obtained to explain the reason. Further, the taxa in Cyathostominae may be redefined based on mt genomes data.

## 5. Conclusions

The present study first reported the complete mt genome of *C. elongatus*, which contained 12 PCGs, 22 tRNAs, 2 rRNAs, and 2 NCRs. Our findings further supported that *C. elongatus* belong to *Cylicocyclus* based on mt genomes. Our results also provide a beneficial reference for taxonomy, population genetics, and systematics studies of Cyathostominae species.

## Figures and Tables

**Table 1:** The gene composition, position, codons, and spacer/overlap regions of complete mt genome of *C. elongatus*.

Genes/Regions	Positions and Sequence Lengths (bp)	Initiation and Stop Codons	Intergenic Nucleotides
*cox*1	1–1578 (1578)	ATT/TAA	0
tRNA-Cys (C)	1579–1634 (56)	–	0
tRNA-Met (M)	1648–1706 (59)	–	13
tRNA-Asp (D)	1707–1765 (58)	–	0
tRNA-Gly (G)	1787–1843 (57)	–	22
*cox*2	1844–2539 (696)	ATT/TAA	0
tRNA-His (H)	2540–2593 (54)	–	0
*rrn*L	2601–3568 (968)	–	7
*nad*3	3572–3907 (336)	ATT/TAA	4
*nad*5	3918–5501 (1584)	ATT/TAG	10
tRNA-Ala (A)	5501–5556 (56)	–	−1
LNCR	5557–5842 (286)	–	0
tRNA-Pro (P)	5843–5897 (55)	–	0
tRNA-Val (V)	5931–5985 (55)	–	33
*nad*6	5986–6420 (435)	ATT/TAA	0
*nad*4L	6473–6706 (234)	ATT/TAA	52
tRNA-Trp (W)	6732–6787 (56)	–	21
tRNA-Glu (E)	6815–6874 (60)	–	27
*rrn*S	6877–7575 (699)	–	2
tRNA-Ser^UCN^ (S2)	7576–7631 (56)	–	0
tRNA-Asn (N)	7630–7684 (55)	–	−2
tRNA-Tyr (Y)	7693–7748 (56)	–	8
*nad*1	7749–8621 (873)	TTG/TAA	0
*atp*6	8632–9231 (600)	ATT/TAA	10
tRNA-Lys (K)	9248–9309 (62)	–	16
tRNA-Leu^UUR^ (L2)	9328–9382 (55)	–	18
tRNA-Ser^AGN^ (S1)	9383–9435 (53)	–	0
*nad*2	9436–10,281 (846)	TTG/TAA	0
tRNA-Ile (I)	10,288–10,346 (59)	–	6
tRNA-Arg (R)	10,374–10,436 (63)	–	27
tRNA-Gln (Q)	10,444–10,498 (55)	–	7
tRNA-Phe (F)	10,505–10,559 (55)	–	6
*cyt*b	10,560–11,672 (1113)	ATT/TAA	0
tRNA-Leu^CUN^ (L1)	11,683–11,737 (55)	–	11
*cox3*	11,738–12,506 (769)	ATT/T	0
tRNA-Thr (T)	12,507–12,564 (58)	–	0
*nad*4	12,565–13,794 (1230)	TTG/TAA	0
SNCR	13,795–13,875 (81)	–	0
Total size (bp)	13,875	–	

Note, “–” is no data.

**Table 2:** The identities of nucleotides sequences of complete mt genomes among 6 *Cylicocyclus* species and *P. imparidentatum*.

Species	Identity Nts (%)						
	*C. el*	*C. as*	*C. i*	*C. au*	*C. n*	*C. r*	*P. i*
Total Size (bp)	13,828	13,817	13,876	13,836	13,831	13,846	13,875
*C. as*	87.1	–	–	–	–	–	–
*C. i*	88.1	88.2	–	–	–	–	–
*C. au*	87.2	87.4	89.6	–	–	–	–
*C. n*	87.1	90.7	88.0	87.2	–	–	–
*C. r*	87.4	90.7	88.0	87.6	89.4	–	–
*P. i*	84.3	82.9	83.6	82.9	83.0	83.3	–

Note, “–” is no data.

## Data Availability

Not applicable.

## Supplementary Materials

The following supporting information can be downloaded at: https://www.mdpi.com/article/10.3390/ani12121571/s1. Table S1: PCR primers used in the amplification of complete mt genomes. Table S2: The gene/region lengths and their amino acids and nucleotides sequence identities of 6 *Cylicocyclus* species and *Poteriostomum imparidentatum*. Figure S1: A + T and G + C contents and skews of 12 PCGs, rRNAs, tRNAs, and complete mt genome sequences of *C. elongatus*. Figure S2: Phylogenetic analyses reconstructed using nucleotide sequences of complete mt genome sequences in 23 Strongylidae species. The tree was developed using ML method. *Oxyuris equi* is an outgroup. *C. elongatus* in current study is underlined.
