# Supplementary material for: The Mitochondrial Genome of Cylicocyclus elongatus (Strongylida: Strongylidae) and Its Comparative Analysis with Other Cylicocyclus Species
Source: Animals (Basel). 2022 Jun 17;12(12):1571. doi: 10.3390/ani12121571 (PMC9219543; doi:10.3390/ani12121571)
Supplement: Supplementary file 1 [file animals-12-01571-s001.zip › Figures S1 and S2.pdf]

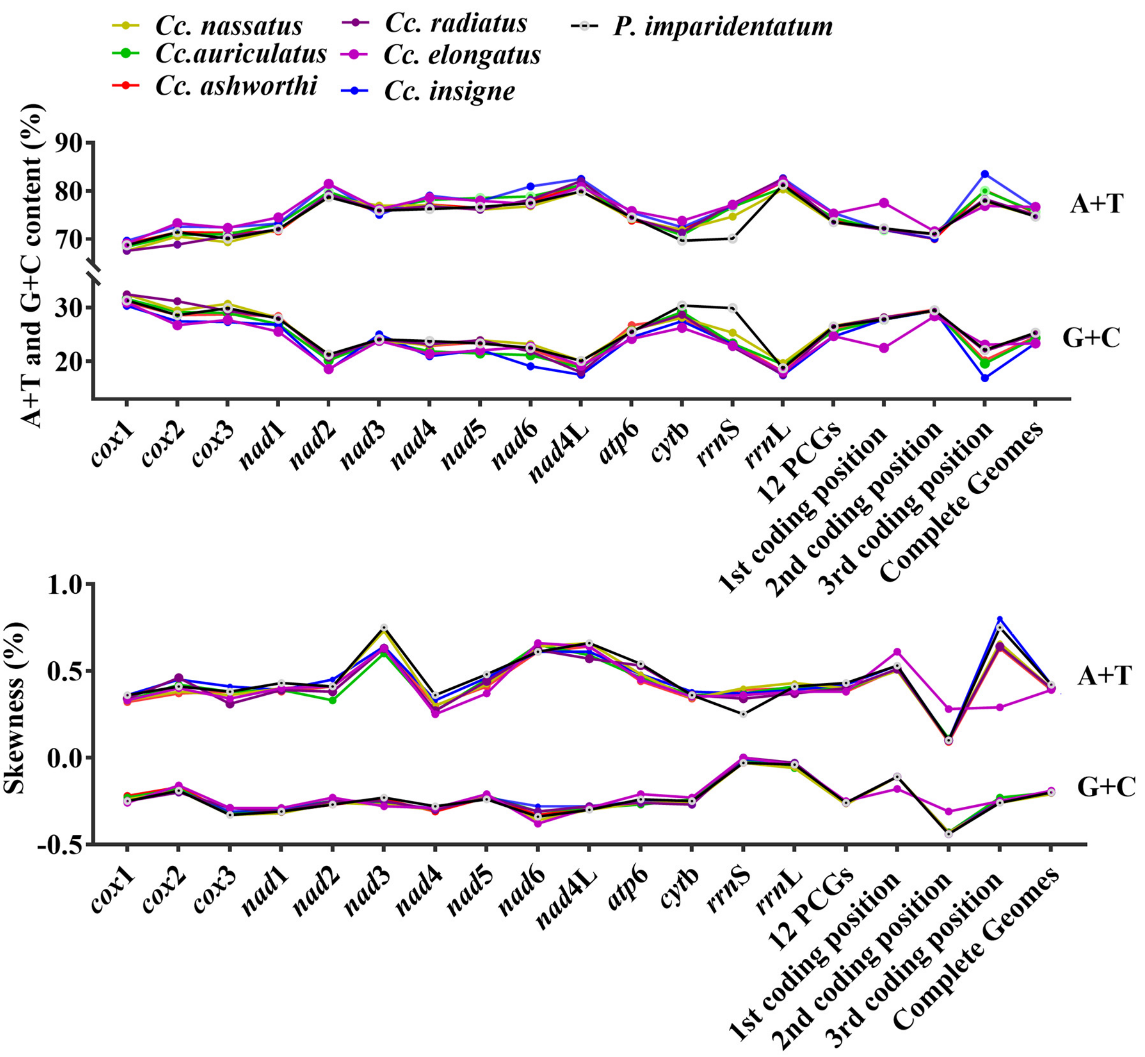

Figure S1. A + T and G + C contents and skews of 12 PCGs, rRNAs, tRNAs, and complete mt genome sequences of *Cc. elongatus*.

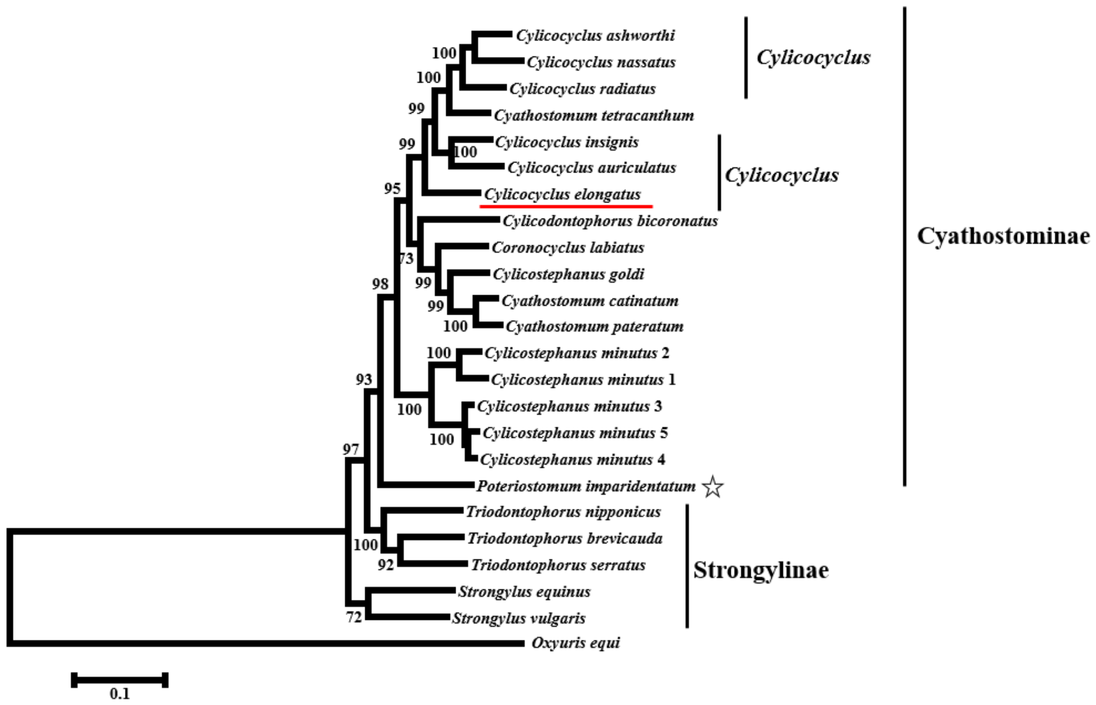

Figure S2. Phylogenetic analyses reconstructed using nucleotide sequences of complete mt genome sequences in 23 Strongylidae species. The tree was developed using ML method. *Oxyuris equi* is an outgroup. *Cc. elongatus* in current study is underlined.
