# Supplementary material for: The Mitochondrial Genome of Cylicocyclus elongatus (Strongylida: Strongylidae) and Its Comparative Analysis with Other Cylicocyclus Species
Source: Animals (Basel). 2022 Jun 17;12(12):1571. doi: 10.3390/ani12121571 (PMC9219543; doi:10.3390/ani12121571)
Supplement: Supplementary file 1 [file animals-12-01571-s001.zip › Table S1.pdf]

Table S1. PCR primers used in the amplification of complete mt genomes.

Primer name	Sequence (5' to 3')
*cox*1-*rrn*L-F	CCATTATTATTATGAGTTTTAGGCT
*cox*1-*rrn*L-R	CCTTAATGTCCTCACGCTAAGACTG
*rrn*L-*nad*5-F	AAAGTTGAAGTATTTTTATAGTTAG
*rrn*L-*nad*5-R	ACCACACAAACAAAACAAAGTAATC
*nad*5-*nad*4L-F	TTTCAAGTGTTGCTGCTATAGTGGA
*nad*5-*nad*4L-R	ATACTAAAGCCTCAAATTCAACTTA
*nad*4L-*atp*6-F	TAAAGTTTTATGGCAGAGATCAGTG
*nad*4L-*atp*6-R	AAGTTCTTAATCATGCTACTATGGC
*atp*6- *nad*2-F	CCTTGTGGAATGGTGGAGTTTACTT
*atp*6- *nad*2-R	CTAATATAGTCTCTCACCCAACTAA
*nad*2- *cox*3-F	TTAATTCTTTCGTCAACGGAATCAT
*nad*2- *cox*3-R	AAAATTACCAACGAGCTTGTCAAAC
*cox*3-*nad*4-F	TTTTTGCTTCTTTGGGTTTGACAAG
*cox*3-*nad*4-R	CATAAAATAATAAATAATAACCTGA
*nad*4- *cox*1-F	TTGTTTGTTTTTTTTATTTCTAGC
*nad*4- *cox*1-R	AATGACTAACCACATAATAAGTATC
