# Supplementary material for: The Mitochondrial Genome of Cylicocyclus elongatus (Strongylida: Strongylidae) and Its Comparative Analysis with Other Cylicocyclus Species
Source: Animals (Basel). 2022 Jun 17;12(12):1571. doi: 10.3390/ani12121571 (PMC9219543; doi:10.3390/ani12121571)
Supplement: Supplementary file 1 [file animals-12-01571-s001.zip › Table S2.pdf]

**Table S2.** The gene/region lengths and their amino acids and nucleotides sequence identities of 6 *Cylicocyclus* species and *Poteriostomum imparidentatum*.

| Genes | *Cc. i* | *P. i* | *Cc. as* | *Cc. r* | *Cc. au* | *Cc. n* | *Cc.el* |
|---|---|---|---|---|---|---|---|
| ***cox*1** | 1,578/525 | 1,578/525 | 1,578/525 | 1,578/525 | 1,578/525 | 1,578/525 | 1,578/525 |
| *P. i* | 86.6/97.3 | – | – | – | – | – | – |
| *Cc. as* | 89.3/97.9 | 86.4/96.4 | – | – | – | – | – |
| *Cc. r* | 88.5/98.5 | 85.4/96.4 | 90.7/98.5 | – | – | – | – |
| *Cc. au* | 89.5/98.9 | 86.2/97.0 | 88.5/97.9 | 88.3/98.5 | – | – | – |
| *Cc. n* | 87.7/98.1 | 86.0/97.5 | 88.9/97.7 | 89.8/97.5 | 88.2/98.1 | – | – |
| *Cc.el* | 89.0/97.9 | 86.2/96.6 | 88.5/97.7 | 87.8/97.9 | 87.2/97.9 | 86.3/97.0 | – |
| ***cox*2** | 696/231 | 696/231 | 696/231 | 696/231 | 696/231 | 696/231 | 696/231 |
| *P. i* | 86.5/97.8 | – | – | – | – | – | – |
| *Cc. as* | 88.8/100.0 | 86.4/97.8 | – | – | – | – | – |
| *Cc. r* | 88.8/100.0 | 87.2/97.8 | 91.2/100.0 | – | – | – | – |
| *Cc. au* | 89.2/99.1 | 86.6/97.4 | 88.1/99.1 | 87.5/99.1 | – | – | – |
| *Cc. n* | 88.4/99.1 | 85.9/97.0 | 90.5/99.1 | 90.2/99.1 | 87.1/98.3 | – | – |
| *Cc.el* | 89.1/99.6 | 86.5/97.8 | 88.4/99.6 | 87.8/99.6 | 89.5/99.6 | 86.9/98.7 | |
| ***rrn*L** | 959/– | 983/– | 978/– | 969/– | 969/– | 974/– | 968/– |
| *P. i* | 83.8 | – | – | – | – | – | – |
| *Cc. as* | 91.9 | 83.5 | – | – | – | – | – |
| *Cc. r* | 91.7 | 83.9 | 94.5 | – | – | – | – |
| *Cc. au* | 90.5 | 83.5 | 90.2 | 91.3 | – | – | – |
| *Cc. n* | 89.6 | 83.0 | 91.4 | 91.7 | 88.6 | – | – |
| *Cc.el* | 91.6 | 83.3 | 91.2 | 90.1 | 89.3 | 89.1 | – |
| ***nad*3** | 336/111 | 336/111 | 336/111 | 336/111 | 336/111 | 336/111 | 336/111 |
| *P. i* | 83.3/92.8 | – | – | – | – | – | – |
| *Cc. as* | 87.5/99.1 | 86.0/92.8 | – | – | – | – | – |
| *Cc. r* | 86.9/98.2 | 84.8/92.8 | 91.7/99.1 | – | – | – | – |
| *Cc. au* | 89.3/99.1 | 84.2/91.9 | 86.0/98.2 | 86.9/97.3 | – | – | – |
| *Cc. n* | 86.9/96.4 | 88.4/93.7 | 87.8/97.3 | 88.7/97.3 | 85.7/95.5 | – | – |
| *Cc.el* | 88.1/97.3 | 84.2/92.8 | 87.2/98.2 | 86.0/98.2 | 85.7/96.4 | 88.7/97.3 | – |
| ***nad*5** | 1,584/527 | 1,584/527 | 1,584/527 | 1,584/527 | 1,584/527 | 1,584/527 | 1,584/527 |
| *P. i* | 86.7/93.5 | – | – | – | – | – | – |
| *Cc. as* | 89.0/96.2 | 86.6/93.7 | – | – | – | – | – |
| *Cc. r* | 87.9/95.6 | 87.4/94.1 | 90.5/97.9 | – | – | – | – |
| *Cc. au* | 90.8/95.3 | 87.0/91.8 | 89.0/94.7 | 89.0/94.9 | – | – | – |
| *Cc. n* | 88.7/96.2 | 86.5/93.7 | 93.4/98.9 | 89.5/97.9 | 88.5/94.9 | – | – |
| *Cc.el* | 88.6/95.3 | 90.3/95.1 | 88.3/95.1 | 88.1/95.1 | 88.8/93.4 | 87.9/94.9 | – |
| **LNCR** | 274/– | 288/– | 270/– | 272/– | 265/– | 270/– | 286/– |
| ***nad*6** | 435/144 | 435/144 | 435/144 | 435/144 | 435/144 | 435/144 | 435/144 |
| *P. i* | 84.8/87.5 | – | – | – | – | – | – |
| *Cc. as* | 88.0/97.2 | 81.8/86.8 | – | – | – | – | – |
| *Cc. r* | 87.4/94.4 | 81.4/86.8 | 89.7/95.8 | – | – | – | – |
| *Cc. au* | 89.0/97.2 | 82.1/88.9 | 89.2/96.5 | 87.6/95.1 | – | – | – |
| *Cc. n* | 89.7/97.2 | 83.2/87.5 | 94.0/98.6 | 87.4/95.1 | 87.1/96.5 | – | – |
| *Cc.el* | 85.5/95.1 | 83.0/85.4 | 85.3/95.1 | 87.4/93.8 | 87.1/94.4 | 86.4/95.1 | – |
| ***nad*4L** | 234/77 | 234/77 | 234/77 | 234/77 | 234/77 | 234/77 | 234/77 |

| | | | | | | | |
|---|---|---|---|---|---|---|---|
| *P. i* | 89.7/94.8 | – | – | – | – | – | – |
| *Cc. as* | 92.3/100 | 87.2/94.8 | – | – | – | – | – |
| *Cc. r* | 92.3/100 | 87.2/94.8 | 95.3/100 | – | – | – | – |
| *Cc. au* | 92.7/100 | 89.3/94.8 | 91.0/100 | 92.3/100 | – | – | – |
| *Cc. n* | 93.6/98.7 | 87.2/96.1 | 93.6/98.7 | 91.9/98.7 | 91.0/98.7 | – | – |
| *Cc.el* | 92.3/100 | 90.2/94.8 | 92.3/100 | 91.9/100 | 91.5/100 | 91.0/98.7 | – |
| ***rrn*S** | 700/– | 709/– | 711/– | 697/– | 699/ – | 699/– | 699/– |
| *P. i* | 79.5/– | – | – | – | – | – | – |
| *Cc. as* | 94.8/– | 79.4/– | – | – | – | – | – |
| *Cc. r* | 95.9/– | 80.1/– | 97.5/– | – | – | – | – |
| *Cc. au* | 95.3/– | 78.7/– | 94.2/– | 94.6/– | – | – | – |
| *Cc. n* | 94.8/– | 78.2/– | 94.6/– | 95.3/– | 93.5/– | – | – |
| *Cc.el* | 95.2/– | 80.1/– | 95.6/– | 96.0/– | 94.4/– | 94.4/– | – |
| ***nad*1** | 873/290 | 873/290 | 873/290 | 873/290 | 873/290 | 873/290 | 873/290 |
| *P. i* | 84.8/94.5 | – | – | – | – | – | – |
| *Cc. as* | 89.0/96.6 | 85.3/91.7 | – | – | – | – | – |
| *Cc. r* | 88.9/98.6 | 86.5/94.1 | 90.7/95.9 | – | – | – | – |
| *Cc. au* | 90.6/97.9 | 85.0/94.1 | 88.2/95.2 | 87.4/97.2 | – | – | – |
| *Cc. n* | 89.6/98.6 | 86.4/94.5 | 89.3/95.9 | 89.3/97.9 | 88.0/97.2 | – | – |
| *Cc.el* | 88.9/97.2 | 86.3/92.8 | 87.6/94.5 | 87.3/96.6 | 89.0/95.9 | 88.2/96.6 | – |
| ***atp*6** | 600/199 | 600/199 | 600/199 | 600/199 | 600/199 | 600/199 | 600/199 |
| *P. i* | 88.3/95.5 | – | – | – | – | – | – |
| *Cc. as* | 90.5/99.0 | 86.8/94.5 | – | – | – | – | – |
| *Cc. r* | 89.0/99.0 | 85.7/94.5 | 90.3/98.0 | – | – | – | – |
| *Cc. au* | 91.3/98.0 | 86.2/93.5 | 88.7/97.0 | 89.8/98.0 | – | – | – |
| *Cc. n* | 88.0/97.5 | 87.3/94.5 | 89.2/96.5 | 89.8/97.5 | 86.5/96.5 | – | – |
| *Cc.el* | 88.5/97.5 | 85.7/94.0 | 87.5/96.5 | 88.5/97.5 | 87.0/96.5 | 88.3/98.5 | – |
| ***nad*2** | 846/281 | 846/281 | 846/281 | 846/281 | 846/281 | 846/281 | 846/281 |
| *P. i* | 80.7/84.0 | – | – | – | – | – | – |
| *Cc. as* | 85.0/89.0 | 80.6/84.0 | – | – | – | – | – |
| *Cc. r* | 85.7/93.2 | 82.7/87.9 | 89.6/94.3 | – | – | – | – |
| *Cc. au* | 87.6/93.6 | 82.2/87.2 | 86.5/92.2 | 88.3/96.4 | – | – | – |
| *Cc. n* | 86.5/93.2 | 81.4/88.3 | 91.3/93.2 | 88.5/96.1 | 87.0/96.1 | – | – |
| *Cc.el* | 87.8/94.3 | 81.4/86.8 | 86.2/91.5 | 87.6/95.4 | 87.5/96.1 | 87.9/95.7 | – |
| ***cyt*b** | 1,113/370 | 1,113/370 | 1,113/370 | 1,113/370 | 1,113/370 | 1,113/370 | 1,113/370 |
| *P. i* | 83.0/89.5 | – | – | – | – | – | – |
| *Cc. as* | 87.8/95.4 | 83.7/90.0 | – | – | – | – | – |
| *Cc. r* | 86.8/95.7 | 83.6/89.5 | 90.8/98.1 | – | – | – | – |
| *Cc. au* | 87.3/95.1 | 82.7/87.8 | 86.4/95.1 | 86.0/95.9 | – | – | – |
| *Cc. n* | 87.4/94.6 | 82.1/88.9 | 90.8/97.6 | 89.9/98.6 | 86.2/95.4 | – | – |
| *Cc.el* | 88.1/94.1 | 83.2/88.9 | 86.8/98.5 | 87.2/96.2 | 86.9/94.9 | 87.2/95.4 | – |
| ***cox*3** | 769/256 | 766/255 | 766/255 | 766/255 | 766/255 | 766/255 | 769/256 |
| *P. i* | 86.1/96.1 | – | – | – | – | – | – |
| *Cc. as* | 87.9/97.7 | 86.0/95.7 | – | – | – | – | – |
| *Cc. r* | 88.6/98.8 | 85.2/96.9 | 90.1/98.8 | – | – | – | – |

| | | | | | | | |
|---|---|---|---|---|---|---|---|
| *Cc. au* | 88.8/97.7 | 84.8/97.3 | 88.4/97.7 | 87.8/98.8 | – | – | – |
| *Cc. n* | 88.7/97.3 | 85.4/96.5 | 88.8/96.5 | 88.7/97.7 | 88.8/97.7 | – | – |
| *Cc.el* | 88.0/98.4 | 86.2/96.1 | 87.6/96.9 | 87.4/98.0 | 87.3/97.7 | 87.1/96.9 | – |
| ***nad*4** | 1,230/409 | 1,227/408 | 1,230/409 | 1,230/409 | 1,230/409 | 1,230/409 | 1,230/409 |
| *P. i* | 85.4/90.2 | – | – | – | – | – | – |
| *Cc. as* | 90.2/97.8 | 83.8/88.8 | – | – | – | – | – |
| *Cc. r* | 89.9/97.8 | 84.4/89.7 | 91.4/97.1 | – | – | – | – |
| *Cc. au* | 90.2/96.3 | 84.6/89.5 | 88.5/95.6 | 89.3/95.6 | – | – | – |
| *Cc. n* | 89.6/98.3 | 84.3/90.2 | 89.6/98.0 | 90.8/97.6 | 88.6/96.1 | – | – |
| *Cc.el* | 90.1/97.3 | 85.1/89.5 | 89.5/96.8 | 89.4/96.6 | 88.5/94.9 | 88.9/97.6 | – |
| **SNCR** | 88/– | 84/– | 82/– | 82/– | 89/– | 79/– | 81/– |
